# Prevalence of Obstructive Sleep Apnea in a High-Risk Population Using the Stop-Bang Questionnaire in Tehran, Iran

**Published:** 2017

**Authors:** Moein Foroughi, Majid Malekmohammad, Amir Sharafkhaneh, Habib Emami, Parisa Adimi, Batoul Khoundabi

**Affiliations:** 1 Chronic Respiratory Disease Research Center, Department of Pulmonary and Sleep Medicine, National Research Institute of Tuberculosis and Lung diseases (NRITLD), Shahid Beheshti University of Medical Sciences, Tehran, Iran; 2 Tracheal Disease Research Center, NRITLD, Shahid Beheshti University of Medical Sciences, Tehran, Iran; 3 Section of Pulmonary and Critical Care Medicine, Baylor College of Medicine, Houston, TX, USA; 4 Tobacco Prevention and Control Research Center, NRITLD, Shahid Beheshti University of Medical Sciences, Tehran, Iran; 5 Clinical Tuberculosis and Epidemiology Research Center, NRITLD, Shahid Beheshti University of Medical Sciences, Tehran, Iran

**Keywords:** Sleep apnea syndrome, Sleep apnea, Obstructive, Epidemiology, Questionnaire, Prevalence, Urban population

## Abstract

**Background:**

Obstructive sleep apnea (OSA) is the most common sleep-related breathing disorder. Despite its significant morbidities and mortality, the majority of patients with OSA remain undiagnosed. The epidemiology of OSA is well studied in Western countries, while there is scarce information on its epidemiology in other countries. We examined the prevalence of high-risk for OSA in a large urban region of Tehran, Iran.

**Materials and Methods:**

We randomly selected 4021 individuals above 18 years in clusters from different districts of Tehran and surveyed them using the Stop-Bang questionnaire. The questionnaire also incorporated the demographic characteristics, education level, history of coronary artery disease and diabetes, and women’s menopausal status. A score of 3 or higher on the Stop-Bang questionnaire indicated the high risk of OSA.

**Results:**

The study population consisted of 2075 (51.6%) females and 1946 (48.4%) males, with the mean age of 40.88 years (SD, 15.4) and mean body mass index (BMI) of 26.18 kg/m^2^ (SD, 4.43). Overall, 51.4% of males and 26.5% of females (total, 1513; 38.6%) were classified in the high-risk group, according to the Stop-Bang questionnaire. The risk of OSA was directly correlated with BMI, advanced age, and history of cardiovascular diseases and diabetes.

**Conclusion:**

According to the Stop-Bang questionnaire, almost 1 out of every 3 individuals was classified in the high-risk group for OSA. Considering the significant morbidity and mortality of this disorder, it is considered a major health problem. Therefore, further detailed studies with confirmatory tests are recommended in order to plan strategies for the diagnosis, treatment, and rehabilitation of these patients.

## INTRODUCTION

Obstructive sleep apnea (OSA) is the most prevalent sleep-related breathing disorder (1). In OSA, the pharyngeal airway collapses repeatedly during sleep and causes complete or partial upper airway obstruction. OSA clinically manifests as loud snoring, choking, or gasping for air during sleep, sleep fragmentation, and excessive daytime sleepiness (2). It is associated with increased mortality and morbidities, including hypertension, cardiovascular diseases, stroke, depression, and anxiety (3–6). Furthermore, it is associated with metabolic diseases, such as diabetes and obesity, besides an increased risk of car accidents (7–10).

OSA is generally an underdiagnosed disorder. Young and colleagues, using the data from the Wisconsin Sleep Cohort, showed that 82% of men and 93% of women with moderate to severe OSA remained undiagnosed (11). The epidemiology of OSA is even more obscure in developing countries due to the limited availability of diagnostic sleep facilities and experts. Therefore, various non-laboratory-based screening tools are used to describe high-risk individuals in these countries.

Two studies conducted in Iran used the Berlin questionnaire to estimate the prevalence of OSA in a high-risk population (12, 13); however, these studies did not include the largest urban area of Iran (Tehran). In the present study, the Stop-Bang questionnaire was applied for screening OSA, as it is a simpler tool in a large cohort of respondents in comparison with the Berlin questionnaire. This questionnaire was first constructed by Chung et al. (2008) as a screening tool for OSA in candidates undergoing surgery (1).

Due to its high sensitivity, straightforward questions, and ease of administration, the Stop-Bang questionnaire has been used successfully as a screening tool in surgical and general populations (14–16). It consists of 8 simple but efficient items for OSA screening, and respondents with 3 (or more) out of 8 items are scored positive. The sensitivity of this test has been estimated at 86.1%, 92.8%, and 95.6% for the apnea-hypopnea index (AHI) > 5, AHI > 10, and AHI > 15, respectively (17); its sensitivity reaches 100% in severe cases of OSA (AHI > 30) (18).

The present study aimed to determine the prevalence of high risk for OSA in a general adult population from Tehran, using the validated Stop-Bang questionnaire.

## MATERIALS AND METHODS

The Ethics Committee of Shahid Beheshti University of Medical Sciences approved this study. The study sample consisted of adults (≥ 18 years) living in Tehran, Iran.

Generally, Tehran, the capital of Iran with 22 municipal districts and a population of around 8.5 million, is the largest city in the country and one of the most populated cities in West Asia. In this study, we applied cluster sampling, with each cluster comprising of 10 households. For appropriate population distribution, 216 clusters were selected from the whole population with respect to each district’s population; clusters from each region were randomly selected. One family was selected as the head of the cluster, from which 10 neighbors were systematically selected in a clockwise order. One male and one female subject, aged above 18 years, were surveyed in each family. In this study, we used the Stop-Bang questionnaire as the survey instrument. In this tool, Stop stands for snoring, tiredness, observed apnea, and blood pressure, as previously validated in the Berlin questionnaire (19). The items were translated to Persian language, and their content validity was approved by an expert panel. To examine the reliability of the Stop section, 12 subjects answered the questions twice in an interval of 10 days, and test-retest reliability coefficient was calculated to be 0.8. The Bang section of the questionnaire includes neck circumference > 40 cm, age > 50 years, body mass index (BMI) > 35, and gender (male). By adding these 4 items to the Stop section, the sensitivity and positive predictive value increased for moderate and severe OSA (1, 14).

Each item in the Stop-Bang questionnaire is scored 1/0 (yes/no questions). A positive result was considered as 3 or more points out of 8 points. The questionnaire was distributed among households. The neck circumference, height, and weight were measured by the interviewers, while the rest of the required information was reported in the questionnaire by the families. In case the participants were illiterate, the questions were asked by the interviewers.

Height was measured by a tape while standing barefoot in an upright position, and weight was measured by a standard digital scale. After each 10 tests, the scales were calibrated by a 5-kg weight. BMI was defined as the individual’s body mass divided by the square of height. The neck circumference was measured by a tape at the neck base. In addition to the Stop-Bang questionnaire, few other questions were added, including the level of education, history of medical conditions (diabetes and coronary artery disease or CAD), and menopausal status of women.

SPSS version 16 was used to analyze the data, and descriptive statistics were calculated. In all tests, *P*-value less than 0.05 were considered statistically significant. A logistic regression model was used to assess the association between high-risk of OSA and age, BMI, medical comorbidities, educational level, and menopausal status in women.

## RESULTS

We enrolled a total of 4021 participants within the age range of 18–97 years (mean, 40.88; SD, 15.4). Overall, 2075 (51.6%) subjects were female and 1946 (48.4%) were male. The mean BMI was 26.18 kg/m^2^ (SD, 4.43); in total, 39.7% of the samples were overweight and 17% were obese. In the study population, 8.1% were diabetic, and 10.2% had CAD. Less than one-third of the participants had a university degree. Table 1 presents the baseline characteristics and comorbidities of the participants. As the findings revealed, 38.5% of the participants reported loud snoring, and 12.7% reported apnea, as observed by their partner.

**Table 1:** Baseline characteristics and comorbidities of the study participants

	***Males***	***Females***	***Total***
**Total number**	1946 (100%)	2075 (100%)	4021 (100%)
**Age categories**			
18–29	510 (26.2%)	483 (23.3%)	993 (24.7%)
30–39	401 (20.6%)	563 (27.1%)	964 (24%)
40–49	354 (18.2%)	425 (20.5%)	779 (19.4%)
50–59	300 (15.4%)	331 (16.0%)	631 (15.7%)
60–69	225 (11.6%)	196 (9.5%)	421 (10.5%)
70+	154 (7.9%)	76 (3.7%)	230 (5.7%)
**BMI categories**			
< 20.00	109 (5.6%)	135 (6.5%)	244(6.1%)
20.00 – 24.99	750 (38.9%)	698 (34.1%)	1448 (36.4%)
25.00 – 29.99	808 (41.9%)	790 (38.6%)	1598 (40.2%)
30.00 – 34.99	216 (11.2%)	331 (16.2%)	547 (13.7%)
35.00 – 39.99	38 (2.0%)	73 (3.5%)	111 (2.8%)
40.00+	5 (0.3%)	20 (1.0%)	25 (0.6%)
**Educational level**			
Illiterate	68 (3.5%)	145 (7.1%)	213 (5.3%)
High school or less	1185 (61.6%)	1367 (66.9%)	2552 (64.3%)
University degree or higher	670 (34.8%)	529 (25.9%)	1199 (30.2%)
**Medical condition**			
Diabetic	142 (7.4%)	182 (8.9%)	324 (8.1%)
Hx of CAD	198 (10.3%)	209 (10.2%)	407 (10.2%)
Hx of hypertension	195 (10.1%)	298 (14.4%)	493 (12.3%)
**Menopausal status**			
Postmenopausal women		523 (29.2%)	
Premenopausal women		1271 (70.8%)	

BMI, body mass index; Hx, history; CAD, coronary artery disease

The complete information of 3923 subjects was available. Overall, 1513 (38.6%) participants were classified in the high-risk group for OSA. Table 2 indicates the number and percentage of high-risk individuals in various subgroups. Loud snoring was the most prevalent symptom in the high-risk group. The prevalence of high-risk for OSA was significantly higher in male participants, compared to females (51.4% vs. 26.5%; *P*> 0.001). Also, the prevalence of high-risk for OSA increased significantly with increasing BMI.

**Table 2.** Baseline Characteristics and Morbidities of High Risk Participants

***Variable***	***High risk for OSA(n)***	***Prevalence rate(%)***	***P value***
**Gender**			
Female	536	26.5	<0.001
Male	977	51.4	
**Age groups**			
18.00– 29.00	142	14.6	<0.001
30.00 – 39.00	204	21.6	
40.00 – 49.00	244	31.2	
50.00 – 59.00	455	73.7	
60.00 – 69.00	310	76.0	
70.00 +	158	71.2	
**BMI groups**			
<20	35	14.5	<0.001
20–25	310	21.7	
25–30	687	43.6	
30–35	369	68.2	
35–40	92	82.9	
40+	20	80.0	
**Educational level**			
Illiterate	122	61.3	<0.001
High school or less	1033	41.3	
University degree or higher	330	28.2	
**Medical condition**			
Diabetic	239	78.6	<0.001
Non-diabetic	1263	35.1	
Positive Hx of CAD	282	71.6	<0.001
Negative Hx of CAD	1224	34.8	
**Menopausal status in women**			
Postmenopausal women	276	54.2	<0.001
Premenopausal women	187	14.9	
**Marital status**			
Married	1318	43.0	<0.001
Single	148	19.3	
Other	30	66.7	

BMI, body mass index; Hx, history; CAD, coronary arytery disease.

The prevalence of high-risk for OSA increased steadily up to the age of 49 and subsequently remained constant with advancing age beyond 50 years. The prevalence of CAD and diabetes was 18.7% and 18.9% in the high-risk group, respectively, while the corresponding rates were 4.6% and 2.7% in the low-risk group. Moreover, the prevalence of high risk for OSA in patients with diabetes and CAD was significantly higher than those without diabetes or CAD. The prevalence of high risk for OSA was higher in postmenopausal women, compared to premenopausal women from the high-risk group. Moreover, the prevalence of high risk for OSA was significantly higher in married subjects, compared to their single counterparts. Also, the prevalence of high risk for OSA was significantly higher in less educated participants.

We used multivariate logistic regression analysis with adjustments for age, sex, BMI, education level, and neck circumference. The adjusted odds ratio (OR) and 95% CI were calculated for different variables, as shown in Table 3. Age, BMI, and history of diabetes and cardiovascular diseases significantly predicted the high risk of OSA. The OR of high risk for OSA was 2.4 times higher in diabetic patients, compared to the nondiabetic group. Also, the OR of high risk for OSA was 2.28 times higher in participants with CAD, compared to those without CAD.

**Table 3.** Estimated Odds Ratios in Multivariate Logistic Regression of OSA risk

	***Odds ratio***	***95% CI***	***P value***
Age 18–29	1		
Age 30–39	1.23	0.92–1.64	0.14
Age 40–49	1.87	1.41–2.50	<0.001
Age 50–59	12.95	9.39–17.86	<0.001
Age 60–69	14.27	9.87–20.64	<0.001
Age >70	9.28	6.02–14.21	<0.001
BMI<20	1		
BMI 20–24.9	1.141	0.705–1.847	0.592
BMI 25–29.9	3.400	2.106–5.489	<0.001
BMI 30–34.9	13.610	8.030–23.068	<0.001
BMI 35–39.9	24.591	11.358–53.241	<0.001
BMI >40	19.242	5.126–72.233	<0.001
Non-diabetic	1		
Diabetic	2.41	1.66–3.52	<0.001
No Hx of CAD	1		
Positive Hx of CAD	2.28	1.66–3.13	<0.001
University degree	1		
High school or less	1.14	0.93–1.41	0.19
Illiterate	1.20	0.76–1.90	0.41

BMI, body mass index; Hx, history; CAD, coronary artery disease

In contrast, education did not have any significant effects. The multivariate logistic regression analysis was applied in the female population with adjustments for age, sex, BMI, education level, and neck circumference, in addition to menopausal status. Similar results were obtained, and menopausal status was discarded as a significant variable (OR, 1.24; *P*= 0.19).

## DISCUSSION

We conducted a survey in a large urban population regarding the high risk for OSA, using cluster sampling. More than 38% of the participants scored 3 or higher on the validated Stop-Bang questionnaire and were at high risk of OSA. Furthermore, the prevalence of high risk for OSA increased with age, BMI, male sex, lower education level, menopausal status (in the female population), and presence of diabetes or CAD. Based on the multivariate logistic regression analysis, age, BMI, male sex, and presence of diabetes or CAD remained significant predictors of high risk for OSA.

The present study is the first epidemiological survey, using the Stop-Bang questionnaire in a general urban population from Iran. An interesting finding of our study is the high prevalence of high risk for OSA in the population. The demographic information of the surveyed sample in our study was similar to previous epidemiological research in Iran (20). In addition, our findings regarding the high prevalence of high risk for OSA are similar to surveys from other large urban regions around the world.

In a study by Adams et al. in Australia, the prevalence of high risk for OSA was 57.1% and 19.3% in men and women, respectively, based on the Stop-Bang questionnaire (14). Similarly, Coelho et al. from Sao Paolo, Brazil reported a corresponding prevalence of 53.57%, according to the Stop-Bang questionnaire (16). A review of studies comparing different questionnaires for OSA screening showed that the Stop-Bang questionnaire has the highest sensitivity, especially in moderate to severe cases (21, 22).

Based on the Berlin questionnaire, which was applied in general populations from the USA, Norway, South Korea, and Saudi Arabia (male population), the prevalence of high risk for OSA was measured to be 26%, 24%, 26%, and 33%, respectively (23–26). In addition, according to this questionnaire, 5% and 27% of the Iranian population were at high risk for OSA in 2 other studies (12, 13); these figures show the lower prevalence of high risk for OSA, which may be associated with the higher sensitivity of the Stop-Bang questionnaire. Furthermore, the prevalence rate reported in our study is only indicative of high risk for OSA, and the percentage of patients with OSA is not determined due to lack of confirmatory tests.

The present study revealed the clear impact of age on the prevalence of high risk for OSA. Age categorization (10-year intervals) facilitated the estimation of increased OSA prevalence. As demonstrated in Table 2, a higher prevalence of OSA symptoms was reported at older age in comparison with the younger age group (18–32 years as the reference age range). Except for the age group of 30–40 years, the difference was significant in other age groups, based on the multivariate regression model. As predicted in people above 50 years, the prevalence of OSA surged remarkably, which is related to the positive point assigned to the question of age in the questionnaire.

Obesity is a main risk factor for OSA and is taken into consideration in various screening tools for OSA. The percentage of obese individuals in our study population (17%) was higher than 2 other epidemiological studies conducted in Iran (8% and 12%) and lower than Brazilian and American studies (21% and 25%) (12, 13, 23, 27). In this study, the prevalence of high risk for OSA increased with higher BMI; the adjusted OR for the group with BMI of 30–35 kg/m^2^ versus the group with BMI < 20 kg/m^2^ was 13.61, which is statistically significant.

In the present study, the prevalence of high risk for OSA was twice higher in men, compared to women. Although male gender was considered as a positive diagnostic point for high risk of OSA, in previous population-based studies, the prevalence of OSA was 1.5 to 3 times higher in males than females (28); this difference could be attributed to the lower airway collapsibility in females with a similar BMI to males (29). Moreover, the risk ratio reduced between males and females (after menopause), which is due to the increased risk among females (28, 30). The multivariate logistic regression analysis of the female population indicated a risk ratio of 1.2 in postmenopausal and premenopausal women after adjustments for the variables (95% CI, 0.8–1.7); despite the higher prevalence, the difference was not statistically significant.

CAD, as the most common cause of mortality worldwide, is also the most fatal comorbidity of OSA. CAD and OSA share common risk factors, such as obesity, male sex, and advanced age. In this study, the prevalence of high risk for OSA among CAD patients was more than 70%. Moreover, 18.7% of subjects from the high-risk group had CAD versus 4.6% of subjects from the low-risk group. Similar to our findings, Peker and colleagues in a 7-year follow-up study reported the incidence of CAD among OSA and non-OSA subjects to be 16.25% and 5.4%, respectively (31).

On the other hand, diabetes is another major comorbidity of OSA with similar risk factors. The present study showed that more than three-fourth of patients with diabetes were at risk of OSA. Also, the prevalence of diabetes was nearly 19% in the high-risk group and 2.7% in the low-risk group; these findings are compatible with previous reports. In this regard, Reichmuth et al., in a large cohort study using polysomnography, reported the prevalence of diabetes to be 14.7% in subjects with AHI > 15 and 2.8% in subjects with AHI < 5 (32). Moreover, Khazaie et al. in their study on a general population from a Western province of Iran reported the prevalence of diabetes to be 15.3% and 2.4% in the high-risk and low-risk groups for OSA, respectively, based on the Berlin questionnaire (13).

One of the limitations of the present study is the absence of confirmatory tests to increase the specificity and accurate estimation of OSA prevalence. Despite the high sensitivity of the applied questionnaire, its specificity in the diagnosis of severe OSA was 37% (21). Therefore, the actual prevalence of OSA in the study population is probably lower than the reported figure. Another limitation of this study is that facial malformations and jaw anatomy, as other contributing factors for OSA, have not been included in the Stop-Bang questionnaire. Moreover, for illiterate subjects, the questionnaire was completed by the interviewers; this intervention might have caused inconsistencies in the Stop-Bang scores and should be considered as another limitation of the present study. Finally, our study showed a high prevalence of high risk for OSA in the selected population from Tehran; therefore, it is not clear whether the findings can be generalized to the inhabitants of other cities of Iran.

Iran, similar to other countries in the region, has insufficient sleep care facilities and sleep specialists. Furthermore, sleep tests are not covered by third-party payers or governmental insurance companies. Also, primary care providers are not familiar with the presentations of OSA, which may result in leaving more patients undiagnosed. Untreated OSA can lead to a nearly two-fold increase in medical expenses, mainly because of cardiovascular morbidity (33). Also, cost-effectiveness of continuous positive airway pressure therapy has been confirmed in patients with moderate to severe OSA (34). Therefore, detection of high-risk individuals and referral for diagnosis and treatment can help improve access to sleep care.

It is suggested to administer the Stop-Bang questionnaire as a proper screening tool in primary healthcare visits to help physicians select further tests. Also, detailed studies using confirmatory tests are recommended to accurately estimate the prevalence of OSA and focus on early detection and preventative methods.
